# Transplacental and Breast Milk Transfer of IgG1 Are Both Required for Prolonged Protection of Offspring Against Influenza A Infection

**DOI:** 10.3389/fimmu.2022.823207

**Published:** 2022-02-03

**Authors:** Julia Chronopoulos, James G. Martin, Maziar Divangahi

**Affiliations:** ^1^ Meakins-Christie Laboratories, Department of Medicine, McGill University Health Centre, Montreal, QC, Canada; ^2^ Department of Microbiology and Immunology and Department of Pathology, Montreal, QC, Canada; ^3^ McGill International TB Center, Montreal, QC, Canada

**Keywords:** pregnancy, influenza A virus, breast milk (colostrum), passive antibody transfer, immunity

## Abstract

The immune system during pregnancy teeters between maintaining fetal tolerance and providing protection against pathogens. Due to this delicate balance, pregnant women and their offspring often have increased susceptibilities to infection. During the first year of life, infant immunity against infection is mainly mediated *via* passively transferred maternal antibodies. However, our understanding of the route of transfer of the maternal antibodies for conferring protection to influenza A virus (IAV) infection in offspring is incomplete. Here we have demonstrated that offspring from IAV-infected mice were significantly protected against IAV infection. This remarkable increase in survival is mediated *via* the elevated maternal serum IgG1. By cross-fostering, we further showed that this enhanced host resistance was only achieved in mice born to and nursed by IAV-infected mothers. Collectively, our data suggest that the prolonged protection of offspring against IAV infection requires maternal IgG1 from both the placenta and breast milk.

## Introduction

Pregnancy has been long thought of as a state of immunosuppression, however, emerging evidence is drawing attention to unique and dynamic processes that differently influence the severity of maternal responses to infectious disease and vaccine outcomes (1, 2). Therefore, strategic intervention during specific gestational periods which support the generation of abundant and efficient pathogen-specific antibodies will protect both the mother and infant against severe illness.

Infants under one year of age are at the highest risk of increased morbidity and hospitalization following infection with pathogens like influenza A virus (IAV), therefore, vaccination is recommended but only beyond 6 months of age (3) (4). During this vaccination gap when children are immunologically immature and harbor a respiratory system that is still developing, maternal immunoglobulins (Ig) are critical for conferring protection against pathogens (5). The magnitude of protection in the offspring is directly correlated with previous maternal exposure to influenza and the concentration of maternal antibodies resulting from natural infection or from vaccination to specific subtypes (with some degree of cross-protection) (5–7). Notably, in humans, maternal vaccination during the second and early third trimester yields higher antibody titers in cord blood and consequently prolongs the window of protection in offspring against influenza and pertussis (8, 9). As a result, public health strategies, especially during the current COVID-19 pandemic, are reinforcing the basis that maternally transferred antibodies can protect newborns from potentially fatal respiratory illness (8, 10, 11).

The lower and upper respiratory tract are kept under surveillance by IgG and IgA, respectively (12). IgG is the most abundant antibody in the serum of which IgG1 and IgG2 subtypes have been implicated in the protection against severe influenza infection in pre-clinical models through their ability to activate complement, neutralize viral particles, and mediating antibody-dependent cell-mediated cytotoxicity (ADCC) and antibody-dependent cellular phagocytosis (13–17). Due to differences in Fc receptor binding and structure, there is a transfer efficiency hierarchy to the neonate among the IgG subclasses with IgG1 being most abundantly transferred (5, 12, 13, 18, 19).

Natural IAV infection is known to trigger longer-lasting antibody responses compared to vaccination (12). Although transplacental maternal antibodies circulate in the infant blood circulation for a limited time, passive immunity continues after birth through nursing where IgA and IgG, are transferred *via* breast milk (5, 12, 20, 21). IgA provides mucosal immunity in the upper airways against IAV infection through comparable mechanisms to IgG in the lungs (12, 22). Though maternal antibodies are known to protect infants from IAV infection (23), whether protection is mediated through transplacental or colostrum IgG and/or IgA and the duration of protection remains unclear. Thus, understanding the underlying mechanism of maternal antibody-mediated protection in offspring can be used for developing vaccine strategies to boost immunogenicity in pregnant women to generate and enhance a specific antibody profile. Using a model of mid-gestation IAV infection and pre-conception infection, we have provided evidence that offspring born to IAV-infected mothers are significantly protected against IAV infection. Furthermore, by cross-fostering, we demonstrated that maternal IgG1 antibodies passively transferred through both the placenta and the colostrum are required to confer the prolonged protection in offspring infected with IAV.

## Materials and Methods

### Mice and Litter Swaps

8-10-week-old female C57BL/6J mice were purchased from Jackson Laboratories, housed, and bred at the animal facility of the Research Institute of the McGill University Health Centre. IAV infections were performed 10 days after mating (E10) following visual identification of a copulation plug or two weeks prior to mating for pre-conception infection. All protocols were approved by the Animal Care Committee of McGill University. In the litter swap experiments, pups were cross-fostered at 3 days old. In the long-term experiments, pupswere weaned at 3-4 weeks and housed by sex. Survival experiments performed on female offspring while all other experiments were performed on both male and females at 6-8 weeks of age.

### Infection

Experiments were performed using the mouse adapted H1N1 influenza virus A/Puerto Rico/8/34 (PR8) virus provided by J.A. McCullers (St. Jude Children’s Research Hospital). The virus was propagated in eggs and titrated in Madin‐Darby Canine Kidney (MDCK) cells by plaque assays. Mice were intranasally challenged with a sublethal dose of 25 or 250 pfu and LD_50_ dose of 500 pfu for survival experiments. Two different stocks derived from the same parental strain were thawed and titrated to determine the LD_50_ and the sublethal doses to be used for experimentation. Viral titers were determined in lung homogenates using standard MDCK plaque assays on days 3 and 6 as previously described (24–27).

### Type I IFN Assays

Total bioactive IFN-α and IFN-β were measured in bronchoalveolar lavage fluid (BALF) and lung homogenates using the murine B16-Blue IFN-α/-β reporter cell line (*In vivo* Gen, San Diego, CA) which monitors the activation of JAK/STAT/ISGF3 and/or IRF3 pathways *via* recognition of IFNAR by type I IFN. The assay was performed according to the manufacturer’s specifications on days 0, 3, and 6 post infection as previously described (24–27).

### Anti-Influenza Antibody Quantification

BALF was collected with a 26-gauge needle though a tracheal cannula using 3 x 1 mL cold PBS and spun at 15,000 rpm for 10 minutes. Lung tissue was homogenized in 500 μl RPMI and spun at 15,000 rpm for 5 minutes. BALF and lung supernatants were stored at -80°C until use. Whole blood was collected by cardiac puncture into microtainer separator tubes (BD # 365967, BD Biosciences, Franklin Lakes, NJ) and was centrifuged at 13,300 rpm for 2 minutes to separate serum. ELISA plates (96 well medium binding microplates, Corning, NY) were coated overnight at 4°C with 2x10^7^ pfu of purified H1N1. Plates were washed and blocked for 2 h with 1% BSA in PBS. Serum, BALF, and lung samples of naïve mice were added, and plates were incubated at room temperature for 2 h. Anti-mouse IgG1 (Southern Biotech #1070-05, Birmingham, AL), anti-mouse IgG2α (Southern Biotech #1081-05), and goat anti-mouse IgA (Southern Biotech #1040-05) secondary antibodies (1:1000) were added. After 2 h incubation at room temperature, reactions were developed with 3,3′,5,5′ tetramethylbenzidine, halted using a sulfuric acid stop solution, and read at 450 nm absorbance on a plate reader.

### Statistical Analysis

Statistical analysis was performed using GraphPad Prism version 9 (GraphPad Software, San Diego, California, USA). All data are presented as mean ± SEM. Statistical differences determined by two-way ANOVA followed by Sidak’s multiple comparison test, Unpaired Student T test or Multiple Student T-tests followed by Bonferroni correction for multiple comparison identified in figure legends.

## Results

### Maternal IAV-Infection Confers IgG1-Mediated Protection to IAV-Infected Offspring

**Figure 1:**
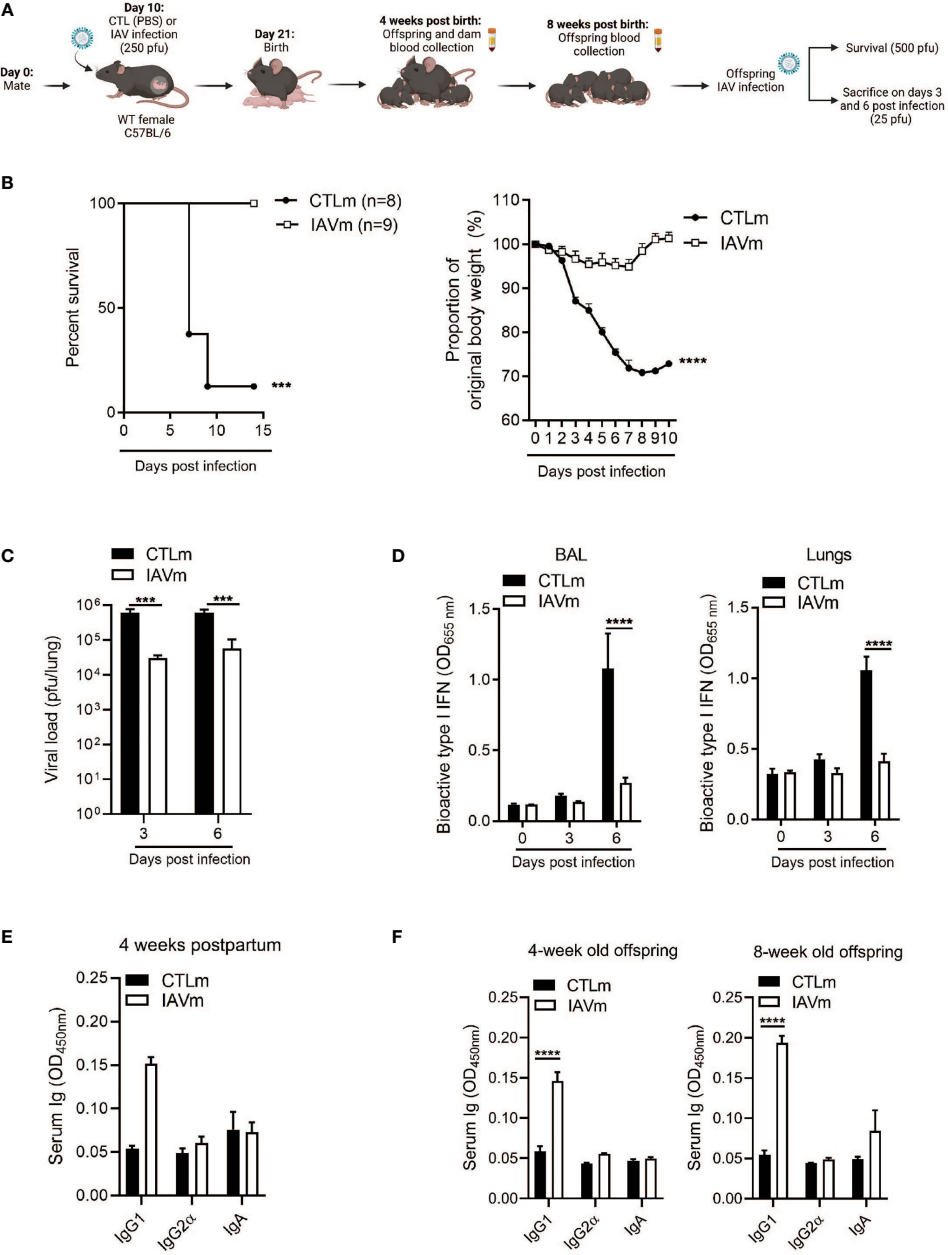
Maternal IAV-infection confers IgG1-mediated resistance to offspring. **(A)** Schematic representation of experimental design. 8–10-week-old C57BL/6 females were given PBS (CTLm; n=2 dams) or infected with 250 pfu A/Puerto Rico/8/34 (PR8) (IAVm; n=3 dams) intranasally on E10. Created with BioRender.com. **(B)** Survival curve and morbidity graph following lethal infection (500 pfu PR8) of 6-week-old females born to control dams (n=8 offspring) or IAV-infected dams (n=9 offspring). **(C)** Kinetics of viral load in whole lung homogenates by MDCK plaque assay and **(D)** type I IFNs in BALF and lungs by B16 assay of 6-week-old offspring infected with 25 pfu PR8 (n=3-5 offspring). **(E)** Serum antibody levels of control and IAV-infected dams 4 weeks postpartum (n=2). **(F)** Serum antibody levels of naïve offspring at 4 weeks and 8 weeks of age prior to secondary infection (n=3-4). Data pooled for male and female offspring. Two-way ANOVA ***p < 0.001, ****p < 0.0001. License acquired for schematics from BioRender.com.

Peak antibody transfer in humans following maternal vaccination occurs in the second and early third trimesters (**Supplemental Figure 1A**), although the exact timing is still unclear (28). Poor placental transfer of maternal antibodies during natural infection with severe acute respiratory syndrome coronavirus 2 (SARS-CoV-2) and influenza virus have been observed in women infected in their final trimester, however, transfer efficiency increases if infection occurs at an earlier gestational period (2, 8, 29, 30). In a mouse model, embryonic day 10 (E10) is the initial phase of type 2 immunity and fetal development in mice (1) and IgG1 antibodies which are the primary neutralizing antibodies during influenza infection are known to be produced in a Th2 environment (12). Therefore, we infected pregnant mice with a sublethal dose of the mouse adapted H1N1 A/Puerto Rico/8/34 (PR8) on E10, mirroring this critical window of pregnancy that allows for optimal and selective antibody transfer (**Supplemental Figure 1A**). Following maternal infection, antibodies were assessed in naïve offspring at 4 and 8 weeks after birth as well as the immune response to secondary IAV infection at 8 weeks (**Figure 1A**). Offspring born to IAV-infected dams had a significant survival advantage following infection with a lethal dose of IAV coupled with negligible weight loss compared to control offspring (**Figure 1B**). Offspring born to IAV-infected dams also showed significantly reduced pulmonary viral burden (**Figure 1C**), which was correlated with a lower induction of type I interferons (IFNs) in the BALF and in the lung tissues (**Figure 1D**).

To determine the link between protection and specific serum antibodies, IgG1, IgG2α, and IgA levels were measured in dams 4 weeks postpartum and in offspring at 4 weeks of age and prior to IAV infection at 8 weeks of age. Serum IgG1 levels were elevated in the IAV-infected dams (**Figure 1E**) and were associated with an elevated serum concentration of IgG1 in naïve offspring at 4 weeks old which was maintained even at 8 weeks of age (**Figure 1F**), for both male and female offspring (**Supplemental Figure 1**).

### Pre-Conception IAV Infection Confers IgG1-Specific Protection to IAV-Infected Offspring

**Figure 2:**
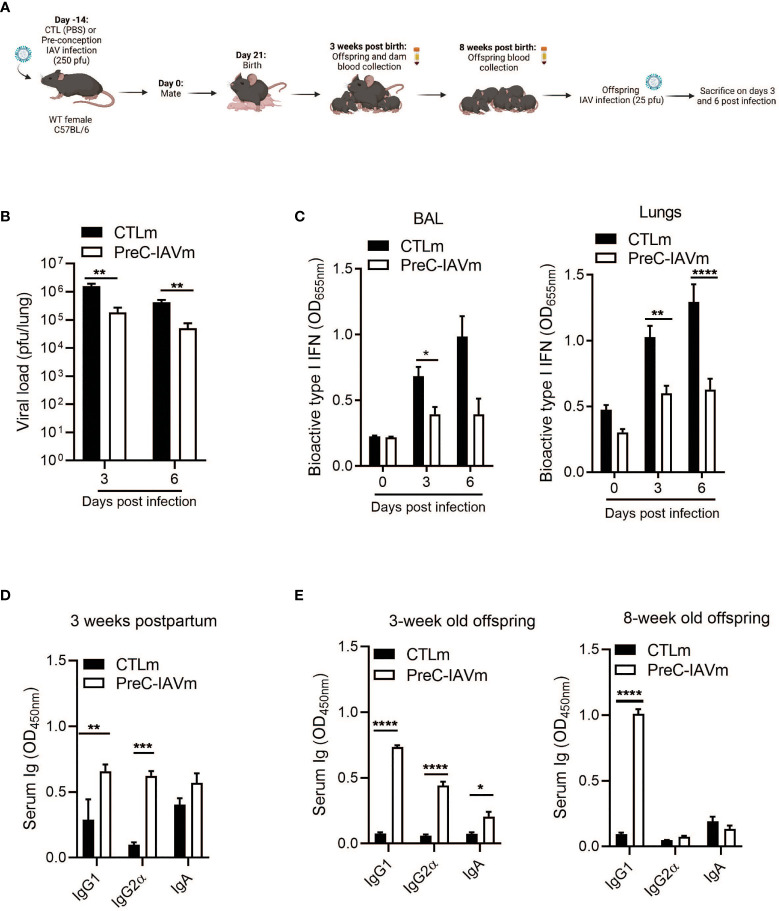
Pre-conception IAV infection confers IgG1-specific protection to offspring. **(A)** Schematic representation of experimental design. 8–10-week-old C57BL/6 females were given PBS (CTLm; n=3 dams) or infected with 250 pfu PR8 (PreC-IAVm; n=4 dams) intranasally two weeks prior to mating. Created with BioRender.com **(B)** Kinetics of viral load in whole lung homogenates by MDCK assay (n=3-4 offspring) and **(C)** type I IFNs in BALF and lungs by B16 assay of 8-week-old offspring born to control or PreC-IAV dams infected with 25 pfu PR8 (n=4-10 offspring). Multiple Student T-tests with Bonferroni correction for multiple comparisons were performed on BALF on day 0 and day 3 *p<0.05. No statistical analysis was performed on day 6 (CTLm n=2). **(D)** Serum antibody levels of control and PreC-IAVm dams 3 weeks postpartum (n=3-4). **(E)** Serum antibody levels of naïve offspring at 3 weeks and 8 weeks of age prior to secondary infection (n=3-7 offspring). Data pooled for male and female offspring. Two-way ANOVA *p < 0.05, **p < 0.01, ***p < 0.001, ****p < 0.0001. License acquired for schematics from BioRender.com.

To determine whether IgG1 antibodies generated against IAV infection prior to pregnancy persist and confer protection to offspring, 8-week-old female mice were infected with IAV two-weeks prior to mating. Offspring from control and pre-conception IAV-infected dams (PreC-IAVm) were then infected with IAV at 8 weeks of age (**Figure 2A**). Offspring born to PreC-IAVm dams had a reduced pulmonary viral burden (**Figure 2B**) with a subsequent reduction in I IFNs in the BALF and lungs (**Figure 2C**). Unlike infection during pregnancy, pre-conception IAV infection generated a broader antibody profile with elevated maternal IgG1, IgG2α, and IgA in the serum 3 weeks postpartum (**Figure 2D**) which mirrored elevated levels in the 3-week-old offspring (**Figure 2E**). However, only IgG1 antibodies were maintained prior to IAV infection in the 8-week-old offspring (**Figure 2E**).

### Antibodies From Placenta or Colostrum Alone Are Insufficient to Confer Protection to IAV-Infected Offspring

**Figure 3:**
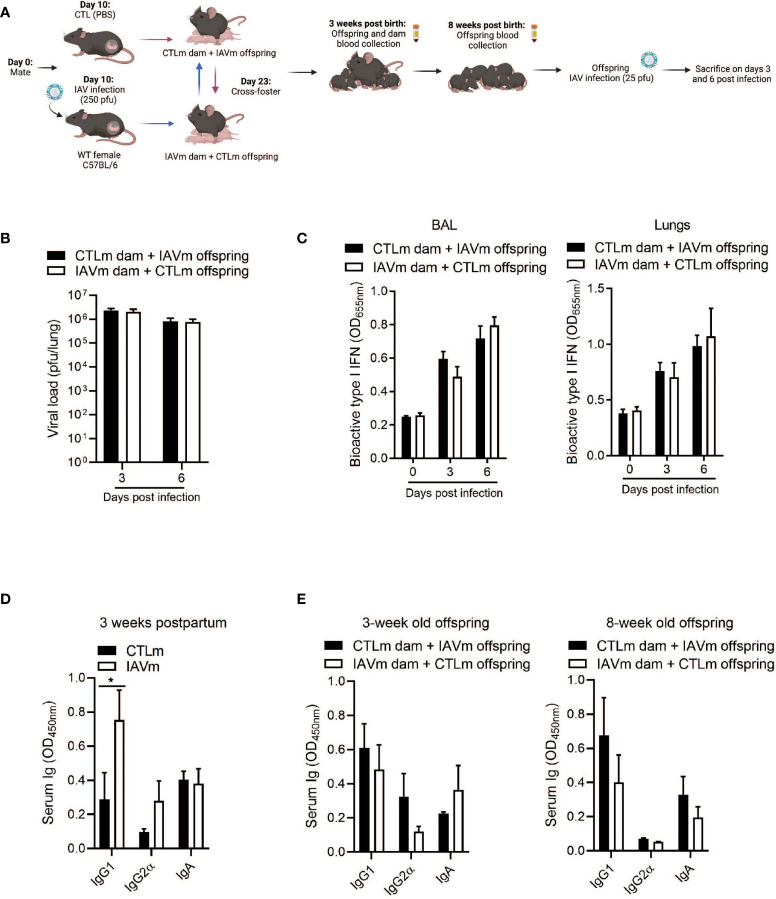
Antibodies transferred solely through placenta or colostrum are insufficient in conferring prolonged protection to offspring against IAV infection. **(A)** Schematic representation of experimental design. 8–10-week-old C57BL/6 females were given PBS (CTLm; n=3 dams) or infected with 250 pfu PR8 (IAVm; n=3 dams) intranasally on E10. Three days after birth, pups were cross-fostered. Created with BioRender.com **(B)** Kinetics of viral load in whole lung homogenates by MDCK assay (n=4-8 offspring) and **(C)** type I IFNs in BALF and lungs by B16 assay of 8-week-old offspring born to IAV dams and fostered by control dams (CTLm dam + IAVm offspring) and of offspring born to control dams and fostered by IAV dams (IAVm dam + CTLm offspring) infected with 25 pfu PR8 (n=4-6 offspring). **(D)** Serum antibody levels of control and IAV-infected dams 3 weeks postpartum (n=3). **(E)** Serum antibody levels of naïve cross-fostered offspring at 3 weeks and 8 weeks of age prior to secondary infection (n=4-6 offspring). Data pooled for male and female offspring. Two-way ANOVA *p<0.05. License acquired for schematics from Biorender.com.

To determine the route of antibody transfer mediating protective immunity, pups from control and IAV-infected dams were cross-fostered. Naïve offspring nursed by IAV-infected dams (IAVm dams + CTLm offspring) and offspring born to IAV-infected dams nursed by control dams (CTLm dam + IAVm offspring) were then infected with IAV at 8 weeks of age (**Figure 3A**). The levels of pulmonary viral load were comparable in both CTLm offspring and IAVm offspring (**Figure 3B**). Similarly, there was no difference in the levels of type I IFNs in the BALF and lungs (**Figure 3C**). Although IAV-infected dams had significantly higher levels of IgG1 compared to control dams (**Figure 3D**), this was not reflected in the offspring at 3 weeks old nor prior to IAV infection at 8 weeks old (**Figure 3E**). There was also no difference in IgA levels detectable in the BALF and lungs of offspring at 8 weeks of age (**Supplemental Figure 1C**). Collectively, these results indicate that the IgG1-mediated protection in IAV-infected offspring required both passive transfer of the antibody from placenta and colostrum of IAV-infected mother.

## Discussion

In our study, passively transferred IgG1 through both the placenta and colostrum provided protection in 6-8-week-old offspring from IAV-infected dams. Studies have shown protection against IAV in the offspring following maternal vaccination from 2 weeks in ferrets (31) to 5 weeks of age in mice, with maternal antibody titers in the murine study declining after 2 weeks of age (7, 16). In our model of mid-gestation IAV infection, we found significant levels of maternal IgG1 in the offspring at 3 weeks old which persisted even at 8 weeks of age. Although antibodies produced after viral infections are predominantly of the IgG2α subtype (9, 14–16, 32, 33), our data are in accordance with previous studies which highlight the critical role of IgG1 during IAV infection (12, 14, 17). While IgG : Fc interactions were not assessed in this study, IgG1 has the longest half-life of about 3 weeks in the newborn and the highest affinity for Fcγ receptors, and thus IgG1 is particularly efficient in neutralizing virus and mediating FcR-dependent effector functions (12).

It has been previously shown that vaccination against pertussis during pregnancy enhances transplacental transfer of IgG1 and subsequent protection to offspring compared to immunization pre-conception (34). In our model, offspring from IAV-infected dams were better protected and presented with a lower pulmonary viral burden from subsequent IAV infection than offspring from PreC-IAVm infected mice, despite the production of a broader antibody profile including IgG1, IgG2α, and IgA in the latter. Although IgG1 is predominantly associated with a Th2 response, it has higher neutralizing abilities against influenza virus compared to the other IgG subtypes (12, 17). While it is not possible to directly compare pregnancy and pre-conception IAV infection due to the differences in immune status of the female, the maternal Th2 environment at E10 could potentially bias antibody production towards IgG1. Thus, the timing of infection during the course of pregnancy can impact the quantity as well as the quality of antibodies produced and differentially modulate the offspring immune system (13). In pertussis infection, it has been shown that Fc glycan modifications during pregnancy allow for the specific placental selection and transfer of NK-activating antibodies mediated by the neonatal Fc receptor and Fc gamma receptor IIIa which provide immunity to offspring (35). In addition, recent studies of vaccination against SARS-CoV-2 highlighted the importance of qualitatively distinct antibody profiles with altered kinetics of pregnant and lactating women compared to the general population (2, 36). Glycan modifications regulate IgG-Fc receptor interactions yielding differences in antibody effector functions and transfer efficiency (9, 12, 37) and are influenced by inflammation, female reproductive hormones, and epigenetic modifications during pregnancy (37–41). Therefore, antibodies generated in a pregnant environment encompass different Fc glycovariants than those generated pre-conception with different binding affinities to specific Fc receptor subtypes and may account for the differences seen in the viral load in their offspring following secondary infection. Thus, Fc glycan profiles and their interactions with placental Fc receptors in the context of IAV infection requires further investigation. Furthermore, in the current study there is a difference in the time elapsed from infection of the mother to the assessment of antibodies in the offspring between infection in pre-conception and during pregnancy. This may also impact the magnitude of antibody subtype produced.

To determine the route of IgG1 transfer conferring protection, pups from control and IAV-infected dams were cross-fostered. Unlike reported protection through the colostrum against infection by H5N1 and H3N2 influenza strains (16, 31, 42), our data suggest that antibody transfer from either nursing or through the placenta alone is insufficient to provide prolonged protective immunity against the H1N1 influenza subtype. The differences in the route, length, and degree of protection among studies may be due to the strain of influenza and the animal model used. Finally, the abundance and contribution of IgG and IgA in mediating protection in these studies could reflect differences in vaccine-induced immunity vs. naturally acquired immunity (36).

## Conclusion

In conclusion, pregnant women and their offspring are vulnerable to both seasonal and pandemic strains of influenza, however, vaccine-elicited immunity and the subsequent transfer of antibodies to the fetus is influenced by the immune status of the mother (2, 9, 29). Boosting maternal IgG1 antibody titers during critical gestational windows in tandem with breast feeding can provide robust and extended protection to offspring against subsequent IAV infection. Understanding the unique immunological milieu during pregnancy and deciphering the maternal-fetal dialogue can guide the development of vaccine regiments to optimize specific maternal antibody profiles and generate long-lasting protection in mother and offspring.

## Data Availability Statement

The raw data supporting the conclusions of this article will be made available by the authors, without undue reservation.

## Ethics Statement

The animal study was reviewed and approved by Animal Care Committee of McGill University.

## Author Contributions

JC performed experiments, analyzed data, and wrote the manuscript with inputs from JM and MD. JC, JM, and MD conceptualized and designed the experiments and discussed the results. JM and MD conceived the project. All authors contributed to the article and approved the submitted version.

## Funding

This work was supported by the Richard and Edith Strauss Foundation.

## Conflict of Interest

The authors declare that the research was conducted in the absence of any commercial or financial relationships that could be construed as a potential conflict of interest.

## Publisher’s Note

All claims expressed in this article are solely those of the authors and do not necessarily represent those of their affiliated organizations, or those of the publisher, the editors and the reviewers. Any product that may be evaluated in this article, or claim that may be made by its manufacturer, is not guaranteed or endorsed by the publisher.

## Supplementary Material

Supplementary Figure 1
**(A)** Schematic comparison of the immunology of pregnancy in humans versus mice. **(B)** IgG1 antibody levels of naïve 6-8-week-old male (n=7-13) and female (n=5-7) offspring born to control (n=5) and IAV-infected dams (n=6) Unpaired Student T-test **p<0.005. **(C)** IgA antibody levels in BALF and lungs of offspring from CTLm (n=9), IAVm (n=3), PreC-IAVm (n=5), CTLm dam + IAVm offspring (n=6), IAVm dam + CTLm offspring (n=5). Unpaired Student T-test **p < 0.005.
