# Supplementary figures and images for: Transplacental and Breast Milk Transfer of IgG1 Are Both Required for Prolonged Protection of Offspring Against Influenza A Infection
Source: Front Immunol. 2022 Feb 3;13:823207. doi: 10.3389/fimmu.2022.823207 (PMC8850295; doi:10.3389/fimmu.2022.823207)

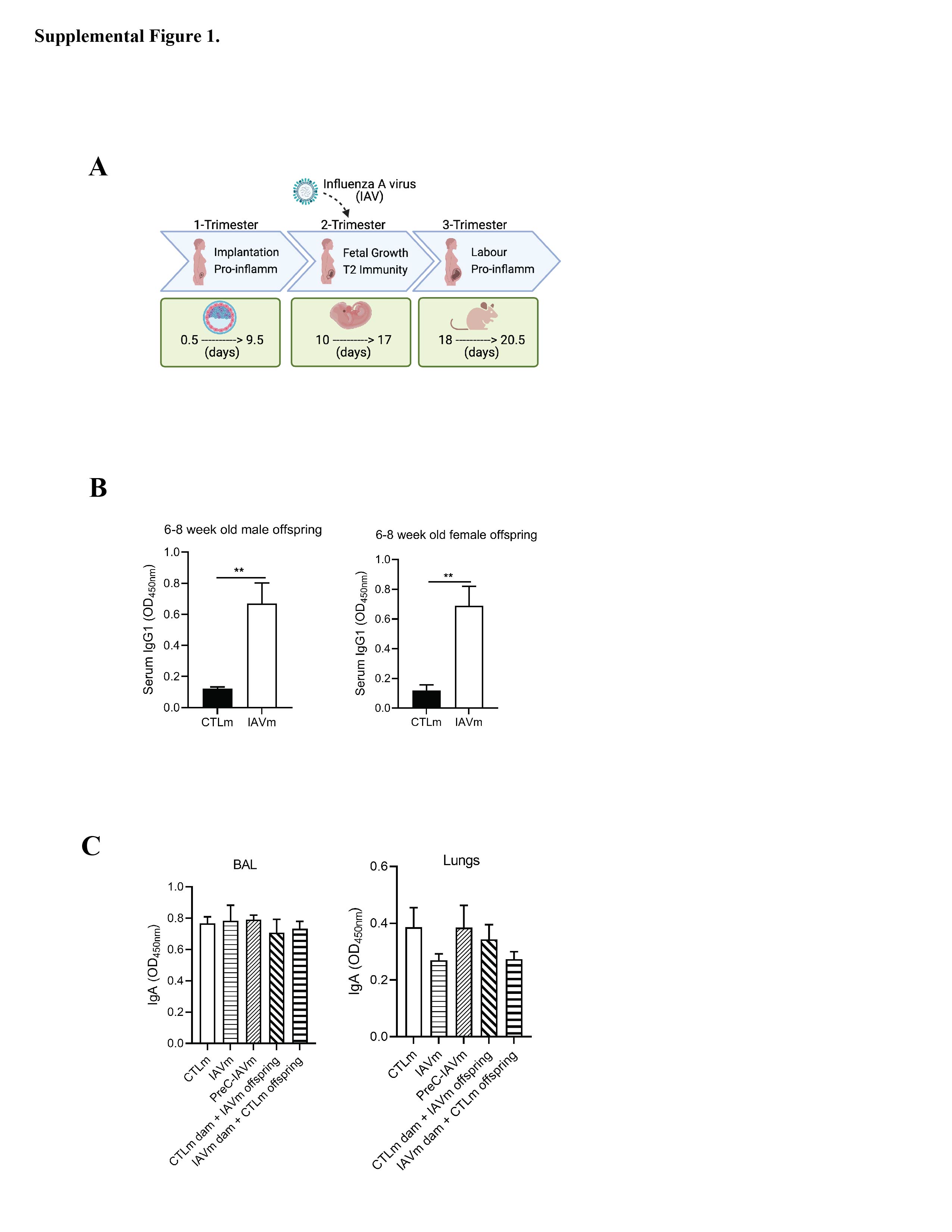

Supplement: Supplementary Figure 1 — (A) Schematic comparison of the immunology of pregnancy in humans versus mice. (B) IgG1 antibody levels of naïve 6-8-week-old male (n=7-13) and female (n=5-7) offspring born to control (n=5) and IAV-infected dams (n=6) Unpaired Student T-test **p<0.005. (C) IgA antibody levels in BALF and lungs of offspring from CTLm (n=9), IAVm (n=3), PreC-IAVm (n=5), CTLm dam + IAVm offspring (n=6), IAVm dam + CTLm offspring (n=5). Unpaired Student T-test **p < 0.005. [file Image_1.jpeg]
